# Preparation and Photoluminescence of Transparent Poly(methyl methacrylate)-Based Nanocomposite Films with Ultra-High-Loading Pendant ZnS Quantum Dots

**DOI:** 10.3390/polym10111217

**Published:** 2018-11-02

**Authors:** Jinku Xu, Dongmei Li

**Affiliations:** Biomaterials and Drug Delivery Laboratory, School of Chemistry and Pharmaceutical Engineering, Qilu University of Technology (Shandong Academy of Sciences), Jinan 250353, China; lidongmei0126@126.com

**Keywords:** nanocomposites, Quantum dots, poly(methyl methacrylate), photoluminescence

## Abstract

Transparent nanocomposite films containing quantum dots are popular because of their extensive applications. However, nanoparticles tend to aggregate, resulting in phase separation of the nanoparticles in the polymer matrix. Herein, we present a bulk thermo-curing copolymerization method to fabricate poly(methyl methacrylate)-based nanocomposite films with ultra-high-loading ZnS quantum dots (ZnS/PMMA), utilizing polymerizable group-capped ZnS and monomer of methyl methacrylate (MMA). We found that the nanocomposite film is highly transparent, although the transmittance decreases with the ZnS content, especially at the wavelength between 300 nm and 400 nm. The results from X-ray diffraction (XRD), transmission electron microscopy (TEM), and dynamic mechanical thermal analysis (DMTA) show that the ZnS quantum dots maintain their original crystal structure, and are uniformly dispersed in the nanocomposite films, even with a very high ZnS content (41 wt %, determined by thermogravimetric analysis). The thermogravimetric analysis shows that the nanocomposite films possess a better thermal stability than that of pure PMMA film. The photoluminescence measurements show that ZnS/PMMA nanocomposite films have good optical properties. The fluorescence intensity increases with the increment of free ZnS content to 30 wt %, and then decreases due to self-reabsorption at a higher ZnS content. The transparent ZnS/PMMA nanocomposite films have a potential application as photoluminescence material.

## 1. Introduction

Quantum dots (QDs) possess attractive properties, such as narrow tunable emission and a high photoluminescence quantum yield (PLQY), making them promising candidates for numerous applications, including light-emitting diodes (LEDs) [1], photodetectors [2], biological labeling and imaging [3,4], lasers [5], and energy harvesting [6]. However, quantum dots are sensitive to moisture and oxygen, and tend to aggregate as a result of their highly specific surface energies. Incorporation of quantum dots in the polymer material can improve its stability, because the aggregation can be blocked by polymers among nanoparticles, and moisture can be prevented by the polymer matrices, especially hydrophobic ones. Moreover, the incorporation of inorganic quantum dots can endow the materials’ new or largely enhanced properties.

Many efforts have been made to develop QDs/polymer nanocomposite films. Quantum dots possess unique size-dependent properties; therefore, it is critical to control the nanoparticle size and size distribution, as well as the dispersion homogeneity in the polymer matrix. Two main approaches have been developed to fabricate nanocomposite films’ incorporated quantum dots, including either in-situ nanoparticle formation [7] or the bulk polymerization of monomer solution to fill premade quantum dots. Obviously, the latter approach is usually more advantageous because of the controllable size and size distribution of premade quantum dots, enlarging their unique size-dependent properties. Unfortunately, quantum dots also tend to aggregate in the process of bulk polymerization, resulting in severe optical scattering and reduced transmittance of the nanocomposites. This will decrease the photoluminescence quantum yield of quantum dots to UV irradiation. To our knowledge, usually only a small amount of quantum dots (usually less than 10 wt %) can be incorporated in the nanocomposite films without sacrificing material properties resulting from aggregation. Researchers used to think that incorporation of a small amount of QDs is enough for photoluminescence, because self-reabsorption usually happens when the content of quantum dots is high in the nanocomposite films. However, according to a recent study by Chao Liu et al. [8], self-reabsorption can be alleviated by adding organic dyes with large Stokes shifts and great absorption at QD emission wavelengths that help extract QD-borne excitons for photon production. They prepared ultra-high-loading quantum/polymer nanocomposites with lower-band-gap organic dyes, resolving the problem of severe light yield deterioration, and the nanocomposite films exhibited simultaneous improvements in both light yield and gamma attenuation. Therefore, it is important to fabricate uniform nanocomposite films with high-loading quantum dots. Moreover, our previous work [9,10] shows that high incorporation of ZnS QDs helps to improve the refractive index of nanocomposites. We grafted polymerizable molecules on the surface of a ZnS quantum dot, and then the high content of ZnS was pendant onto the polymer backbone by copolymerization with monomers of 2-hydroxyethyl methacrylate (HEMA) and N,N-dimethylformamide (DMA). The true ZnS content determined by thermogravimetric analysis can be improved to 50 wt %, and the nanocomposite is high transparent. The incorporation of the high content of ZnS greatly improves its refractive index.

Poly(methyl methacrylate) (PMMA) is a key polymer component for nanocomposite films, with wide applications in many technological and productive fields, which take advantage of its unique properties, including excellent transmittance from the near UV to the near IR [11], good mechanical properties, thermal stability, low dielectric constant, and moisture resistance. In particular, its moisture resistance can block the contact of water to quantum dots in the nanocomposite, making it more stable. Low UV absorption of a PMMA matrix helps more energy be absorbed by quantum dots under UV irradiation, maintaining the photophysics associated with the photoexcited states of the crystallites in the nanocomposites.

Many kinds of quantum dots—e.g., ZnO [12], CdTe [13], ZnS [14], and (CdSe)ZnS [15]—have been incorporated in PMMA-based materials, either by being embedded physically in the polymer matrices by weak interactions, through hydrogen bonding or Van der Waals interactions, or strongly linked through the formation of covalent or ionic bonds with polymer backbones [16]. However, the nanoparticle content in the nanocomposites is usually low, and it is still a huge technological challenge in the fabrication of transparent PMMA-based nanocomposites with high quantum dot content. In this work, we grafted polymerizable molecules onto the surface of a quantum dot of ZnS, and then prepared high-ZnS-loading, PMMA-based nanocomposites by bulk copolymerization between polymerizable group-capped ZnS and MMA monomers. The ZnS nanoparticle can be dispersed uniformly in the nanocomposite films, resulting in the ZnS/PMMA nanocomposite films being highly transparent, even with ultra-high-loading ZnS. The effect of ZnS incorporation into the nanocomposites on the material characterization was also studied, indicating a potential application as photoluminescence material.

## 2. Experimental Part

### 2.1. Materials

Zinc acetate dihydrate and thiourea were purchased from Damao Chemicals (Tianjin, China). Mercaptoethanol (ME), 2-isocyanatoethyl methacrylate (IEM), and dibutyltin dilaurate (DBTDL) were purchased from Aladdin Chemical Reagent Co., Ltd. (Shanghai, China). Methyl methacrylate (MMA) and azobisisobutyronitrile (AIBN) were purchased from SA Chemical Technology Co., Ltd. (Shanghai, China).

### 2.2. Synthesis of Polymerizable Group-Capped ZnS Nanoparticles

Polymerizable-group-capped ZnS nanoparticles (NPs) were synthesized according to our previous work [9]. Briefly, Zn(Ac)_2_·2H_2_O (11.0 g, 0.05 mol), ME (5.8 g, 0.074 mol), thiourea (2.75 g, 0.036 mol), and DMF (150 mL) were added to a 25 mL three-necked round-bottom flask equipped with a magnetic stirrer, a condenser, and nitrogen purging. The solution was refluxed at 160 °C for 10 h under continuous stirring and nitrogen purging. The resultant mixture was concentrated and then precipitated, using excess ethanol to obtain ME-capped ZnS nanoparticles. ME-capped ZnS in the amount of 3 g and one drop of DBTDL were dispersed in 75 mL DMF solution to obtain a clear solution, in which a DMF solution of 2-isocyanatoethyl methacrylate (IEM) (0.45 g in 15 mL DMF) was added into the DMF solution of ME-capped ZnS nanoparticles drop by drop by a constant pressure drop funnel. The solution was kept at 30 °C for 2.5 h, and then precipitated in excess methanol. The solid precipitation (named as ZnS_0.15_, as the subscript of 0.15 corresponds to the 0.15 g amount of added IEM per gram of ME-capped ZnS) was collected and washed with methanol before being dried in vacuum at 30 °C.

### 2.3. Preparation of Transparent ZnS/Poly(methyl methacrylate) Nanocomposite Films

The ZnS/PMMA nanocomposite films were prepared by a bulk thermo-curing copolymerization approach. Firstly, ZnS_0.15_, with the formulations as described in Table 1, was dispersed in DMF by ultrasound, and then the monomer of MMA and 0.5 wt % initiator of AIBN were added to obtain homogeneous polymerization solution. Finally, the polymerization solution was injected into the cavity of the polypropylene plate mold, separated by a polypropylene frame with a controlled thickness, and then cured at 80 °C for 12 h to obtain transparent PMMA-based nanocomposite films.

### 2.4. Characterization of ZnS/Poly(methyl methacrylate) Nanocomposite Films

#### 2.4.1. X-ray Diffraction

X-ray diffraction (XRD) data of ME-capped ZnS, polymerizable group-capped nanoparticles, and ZnS/PMMA nanocomposites were recorded on a Bruker AXS D8 X-ray diffractometer (Rheinstetten, Germany) with a Cu Kα (λ = 1.5406 Å) source, operated at 40 kV and 20 mA at 293 K. The 2θ scanning range was from 10° to 90°, with a step size of 0.02° and at a scanning speed of 1 degree/min.

#### 2.4.2. Transmission Electron Microscopy

ZnS/PMMA nanocomposite films were embedded in Epon 812 epoxy resin, and then polymerized overnight at 60 °C. Ultrathin sections were cut using a Boeckeler Power Tome XL microtome (Tucson, AZ, USA). Cross-sections of the nanocomposite films were observed and photographed using a JEM-2100 transmission electron microscope (Tokyo, Japan). ME-capped ZnS and polymerizable group-capped nanoparticles were also observed with JEM-2100.

#### 2.4.3. Dynamic Mechanical Thermal Analysis

The nanocomposite films were cut into 5 × 30 mm^2^ rectangular bars, and their dynamic mechanical behaviors were recorded on a Tritec 2000 (Hayward, CA USA) instrument in a single cantilever bending mode. A storage modulus (E’) and tan δ were registered as a dependence on temperature ranging from 0 to 250 °C, using a 5 °C/min heating rate at a frequency of 1 Hz.

#### 2.4.4. Thermogravimetric Analysis

ME-capped ZnS, polymerizable group-capped ZnS NPs, as well as the nanocomposite films in the dry state were sealed in aluminum pans, and then thermogravimetric analysis (TGA) was performed using a SDT-Q600 TGA instrument (New Castle, DE, USA) at a heating rate of 10 °C/min in the temperature range from ambient to 600 °C in a nitrogen gas purge.

#### 2.4.5. Light Absorption/Transmittance Spectra

ME-capped and polymerizable-group-capped ZnS were dispersed in DMF under ultrasound to obtain a clear and uniform solution with a concentration of 10 mg/mL. The nanocomposite films were cut into 10 × 40 mm^2^ strips and placed vertically in the light road. Transmittance and absorption of the nanocomposite films were recorded at wavelengths from 200 to 800 nm with a UV-Vis spectrophotometer (Heλios, Thermo Electro, Cambridge, UK), using the air as a reference. The transmittance and absorption of ME-capped and polymerizable group-capped ZnS were determined using DMF as a reference at the same wavelength range.

#### 2.4.6. Photoluminescence Spectra

Photoluminescence (PL) spectra of ME-capped and polymerizable group-capped ZnS NPs and ZnS/PMMA nanocomposite films were measured on a VARIAN Cary Eclipse fluorescence spectrophotometer (Lake Forest, CA, USA) with an Xe-lamp as the excitation source, the tube voltage at 600 V, and excitation and emission slits of 5 nm.

## 3. Results and Discussion

**Scheme 1:**
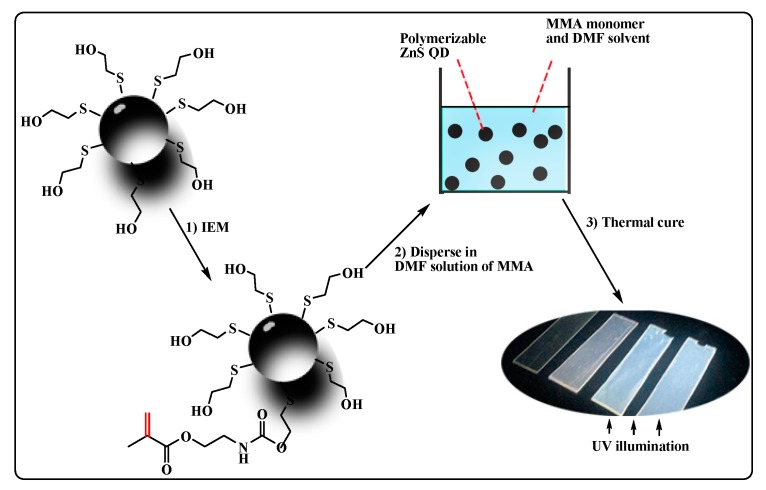
Schematic illustration of the partial chemical graft of polymerizable molecules on the mercaptoethanol (ME)-capped ZnS surface and the bulk copolymerization process of poly(methyl methacrylate)-based nanocomposite films with ultra-high-loading ZnS quantum dots (ZnS/PMMA). IEM is an abbreviation of 2-Isocyanatoethyl methacrylate, and MMA means a monomer of methyl methacrylate. The last photograph is ZnS/PMMA nanocomposite films under UV illumination (the thickness is 0.7 mm, with a loading free ZnS_0.15_ quantum dots (QDs) varying from 10 wt % to 70 wt %, at a 20 wt % increment from left to right).

**Figure 1:**
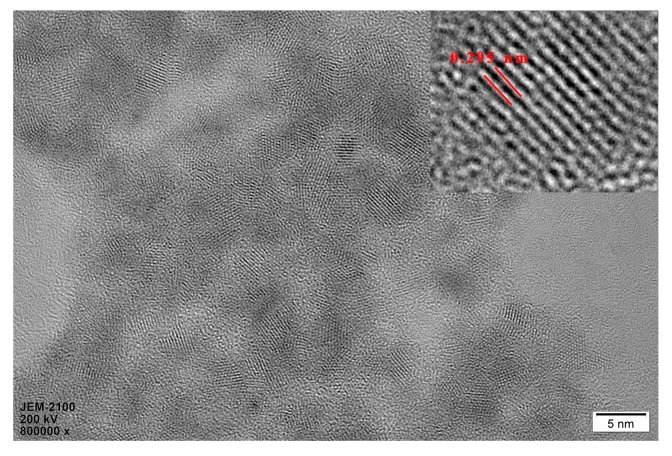
High-resolution transmission electron microscopy (HRTEM) photography of polymerizable group-capped ZnS_0.15_.

The synthesis route of transparent high QD-loading nanocomposite films was shown in Scheme 1. A multi-hydroxyl group on the surface of ME-capped ZnS, from tightly absorbing mercaptoethanol, endows grafting sites for polymerizable molecule. Polymerizable group-capped ZnS was synthesized by a partial chemical graft of the hydroxyl on the surface of ZnS by 2-isocyanatoethyl methacrylate. ME-capped and polymerizable group-capped ZnS can be well-dispersed in DMF to obtain a clear solution. High-resolution transmission electron microscopy (HRTEM) photography, as shown in Figure 1, indicates that the nanoparticle diameter is about 3 nm with a uniform size distribution. The lattice fringes with a distance of 0.294 nm can be clearly observed, indicating that the grafting reaction of the polymerizable molecules on the ZnS surface do not influence the crystalline structure of ZnS NPs. This is important for the quantum dot to maintain its attractive properties, such as tunable emission, high photoluminescence quantum yield, and high refractive index. According to Rayleigh’s law, light scattering of the spherical particles in the nanocomposite films leads to an intensity loss that can be calculated as follows:

(1) $$I=I_{0}\exp[-\frac{3\phi lR^{3}}{4\lambda^{4}}(\frac{n_{NP}}{n_{p}}-1)]\;$$

where *I*_0_ and *I* are the intensity of the incident and transmitted light, respectively; *ϕ* is the volume fraction of the particles; *l* refers to the thickness of the nanocomposite film; and *λ* is the wavelength of the incident light. The variables *n_NP_* and *n_P_* are the refractive index of nanoparticles and the polymer matrix in the nanocomposite films, respectively. It can be concluded from Equation (1) that the size of the nanoparticles in the nanocomposite films has a much stronger effect on the transmittance than the film thickness. Generally, the upper limit of the nanoparticle size is about 40 nm for transparent nanocomposite film, to avoid the loss of transmitted light from scattering. The refractions of PMMA and quantum dot ZnS are about 1.5 and 2.3, respectively. The volume fraction of the ZnS nanoparticles can be calculated by the weight percent of added ZnS_0.15_ in the nanocomposite, capping the amount of ME on the surface of the ZnS (37.05 wt %) and the densities of pure ZnS (4.1 g cm^−3^), the capping agent of ME (1.11 g cm^−3^), and PMMA matrices (1.18 g cm^−3^). The volume fraction of ZnS NPs in the nanocomposite with 70 wt % content of ZnS_0.15_ is about 27%, and the thickness of the nanocomposite film is about 700 μm. It can be predicted that less than 1% of the incident light intensity will be lost because of the scattering of ZnS NPs in the nanocomposite films. Therefore, nanoparticle aggregation will be a considerable factor affecting transmittance of nanocomposite films.

**Figure 2:**
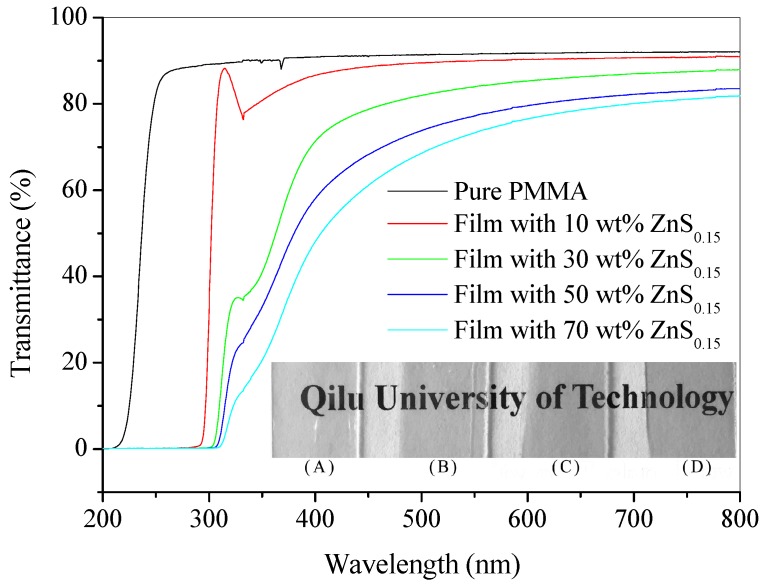
Transmittance spectra of ZnS/PMMA nanocomposite films, with different free ZnS_0.15_ content. The inserted photograph is the appearance of ZnS/PMMA nanocomposite films with a free ZnS_0.15_ content of (**A**) 10 wt %, (**B**) 30 wt %, (**C**) 50 wt %, and (**D**) 70 wt % (sample thickness: 0.7 mm).

The solvent of DMF was used in the preparation of the nanocomposite films, because polymerizable group-capped ZnS nanoparticles cannot disperse in the monomer of MMA. In the bulk thermo-curing copolymerization strategy, all reagents were mixed together as follows: polymerizable ZnS_0.15_ was dispersed in the DMF solvent, and then the monomer of MMA and initiator of AIBN were added under ultrasonic dispersion to form a clear and uniform polymerizable solution. After 12 h curing at 80 °C, the nanocomposite films were transparent, as shown in the inserted photograph in Figure 2. We also prepared a nanocomposite film by physically embedding ME-capped ZnS NPs, which were opaque, even decreasing the ME-capped ZnS less than 5 wt %, although the polymerizable solution was also clear and uniform. This indicates that the difference between ME-capped ZnS nanoparticles and the MMA monomers was enlarged during the polymerization of monomers into a polymer. The attachment of ZnS NPs onto the polymer backbone by copolymerization between polymerizable ZnS_0.15_ and the monomer of MMA blocks the microphase separation of ZnS NPs from the PMMA matrix, to which the enlarged difference can be ascribed.

Pure PMMA film shows high transparency between 250 and 800 nm; while the nanocomposite films with high ZnS content are transparent, the incorporation of ZnS in the nanocomposite films decreases its transmittance, especially at the wavelength ranging from 300 nm to 400 nm, as shown in Figure 2. This indicates that the ZnS nanoparticle in the nanocomposite films has a strong absorption. The transmittance of the nanocomposite film with a free ZnS_0.15_ content of 10 wt %, 30 wt %, 50 wt %, or 70 wt % is respectively about 90%, 84%, 77%, or 74% at the wavelength of 555 nm, which is the most sensitive wavelength for human eye and corresponds to yellow and green light [17]. This indicates that ZnS nanoparticles disperse uniformly in the nanocomposite films. However, it seems that the transmittance is much less than that predicted by Rayleigh’s law, indicating possible slight aggregation of ZnS NPs in nanocomposite film with high content of ZnS.

**Figure 3:**
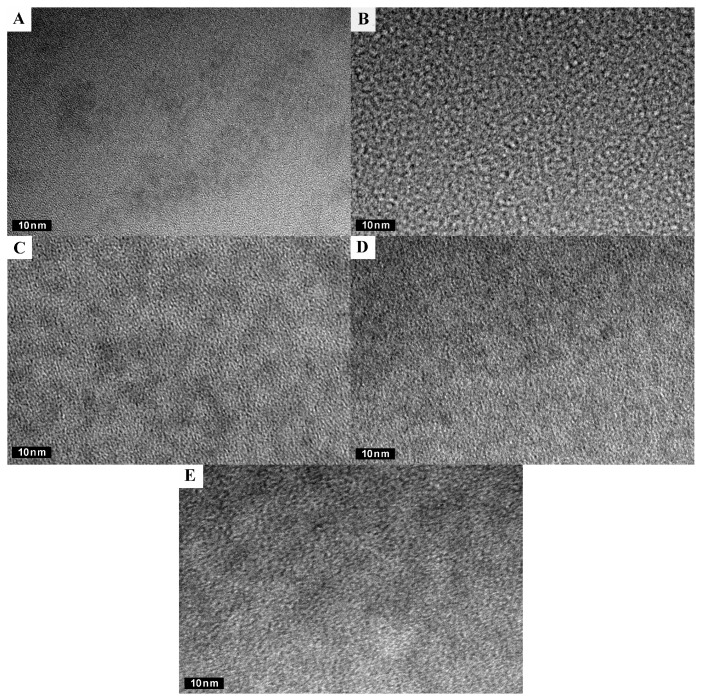
Transmission electron microscopy (TEM) photography of (**A**) ZnS/PMMA nanocomposite films embedded physically into ME-capped ZnS and ZnS/PMMA co-polymerizable nanocomposite films with the following different content levels of free ZnS_0.15_: (**B**) 10 wt %, (**C**) 30 wt %, (**D**) 50 wt %, and (**E**) 70 wt %.

To get further information on the dispersibility of ZnS NPs in the nanocomposites, the ultrathin sections of the nanocomposite films were observed using transmission electron microscopy (TEM). The nanocomposite film embedded in physically ME-capped ZnS NPs clearly exhibits serious particle aggregation, resulting in irregular phase separation, as shown in Figure 3A. Phase separation will cause optical scattering and reduce transmittance, in good agreement with the opaque appearance of ME-capped ZnS embedded physically in nanocomposite film. The TEM photographs of the nanocomposite films from copolymerization between 10–70 wt % polymerizable group-capped ZnS_0.15_ and the monomer of MMA were shown in Figure 3B–E. Although no obvious ZnS NP was observed in the nanocomposite film with 10 wt % ZnS_0.15_, ZnS NPs can be clearly observed when the free ZnS_0.15_ content increased to 30 wt %. It can be seen that the nanocomposite films copolymerized from ZnS_0.15_ and the monomer of MMA can remarkably block ZnS aggregation in resulting nanocomposites. However, slight aggregation of ZnS NPs can still observed, resulting from the phase domain (about 6 nm) that is larger than the size of ZnS quantum dot, as shown in Figure 3C. Figure 3D–E shows a higher nanoparticle content, and the phase domain from aggregation seems to be bigger as the ZnS content increases in the nanocomposite films. This can be seen clearly in Figure 3E, in good agreement with the decreased transmittance shown in Figure 2.

**Figure 4:**
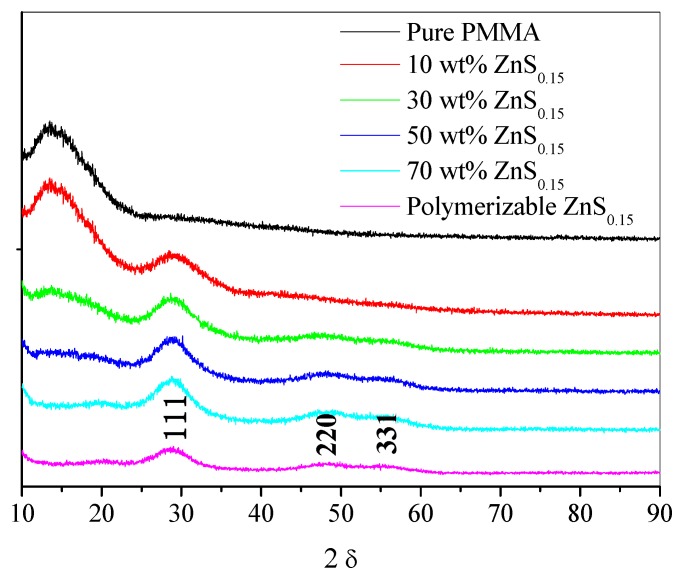
XRD patterns of polymerizable ZnS_0.15_, pure PMMA film, and the nanocomposite films with different ZnS_0.15_ content.

The X-ray diffraction (XRD) patterns of polymerizable ZnS_0.15_ and the nanocomposite film with different content ZnS_0.15_ are shown in Figure 4. It can be seen that three diffraction peaks are observed at 28.5°, 47.6°, and 56.4°, corresponding to the (111), (220), and (311) planes of ZnS with a zinc blend structure [18]. This indicates that the crystal structure and crystallinity are not affected by the grafting reaction of polymerizable molecules on the surface of ME-capped ZnS NP and the copolymerization reaction between polymerizable ZnS_0.15_ and the MMA monomer. The size of the ZnS nanoparticle in the nanocomposite film is also about 3.0 nm, calculated by Scherrer’s formula (d = kλ/βcosθ) based on the (111) peak, in good agreement with the nanoparticle size observed from TEM. Moreover, the nanocomposite films show another broad peak at 13° from PMMA matrices, and the peak intensity rapidly decreases with improved ZnS content in the nanocomposites.

**Figure 5:**
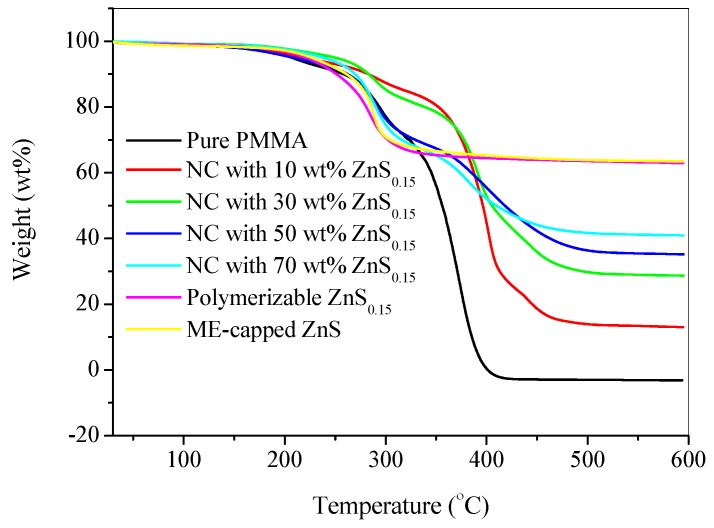
Thermogravimetric analysis (TGA) curves of ME-capped ZnS, polymerizable ZnS_0.15_, and PMMA-based nanocomposite (NC) films with different ZnS_0.15_ content. Heating rate is 10 °C min^−1^ under N_2_ flow.

To determine the actual content of pure inorganic ZnS and the thermal stability of ZnS/PMMA nanocomposite films, thermogravimetric analysis of nanocomposite films with different ZnS_0.15_ content was carried out, compared with the pure PMMA polymer, as well as the ME-capped and ZnS_0.15_ NPs. TGA curves are shown in Figure 5, and the weight loss at each decomposition step and the residue at 600 °C are listed in Table 2. Pure PMMA polymer shows a single degradation step at the temperature range of 260–415 °C, owing to the thermo-decomposition of the PMMA polymer, while the ZnS/PMMA nanocomposite films show two obvious degradation steps corresponding to temperature ranges 250–330 °C and 360–470 °C, respectively. ME-capped ZnS and polymerizable ZnS_0.15_ show a weight loss at the temperature range of 250–330 °C, similar to the first decomposition temperature range of the nanocomposites, due to the decomposition of the ME molecule on ZnS surfaces. Therefore, the second temperature range of weight loss should be ascribed to the decomposition of the PMMA component in the nanocomposite films, indicating improved stability of ZnS/PMMA nanocomposite films. This may be explained in that the interaction between the ZnS nanoparticle and the PMMA polymer matrix is so strong that the thermal degradation of the PMMA polymer is more difficult [19]. The content of pure inorganic ZnS in ME-capped and polymerizable ZnS_0.15_ quantum dots is 62.8 wt % and 63.6 wt %, respectively, indicating the presence of many capping molecules of mercaptoethanol on the nanoparticle surface; only a small number of capping molecules were grafted by the polymerizable molecule. The residue of the nanocomposite films (pure ZnS) with free ZnS_0.15_ content of 10 wt %, 30 wt %, 50 wt %, and 70 wt % is respectively about 13 wt %, 28.7 wt %, 35.2 wt %, and 41 wt %, in good accordance with the calculated values based on the TGA results of the ZnS_0.15_ nanoparticle.

**Figure 6:**
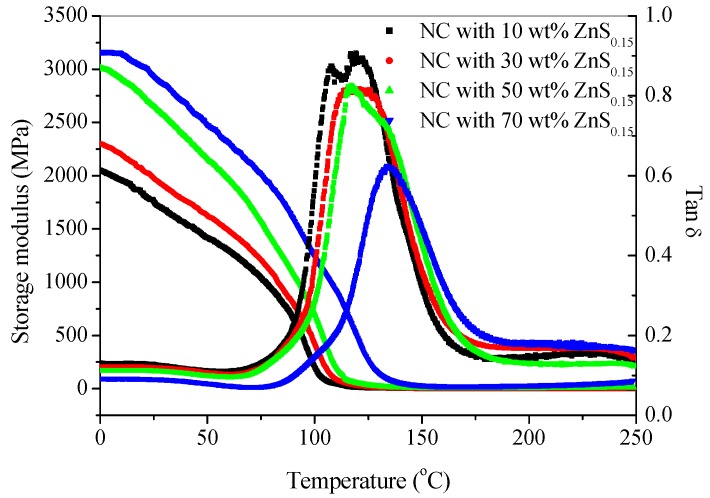
Storage modulus and loss factor (tan δ) of ZnS/PMMA nanocomposite films with different ZnS_0.15_ content.

The storage modulus (*E*) and the loss factor (tan δ) of ZnS/PMMA nanocomposite films with different ZnS content were recorded as a function of temperature. From the viscoelastic curves shown in Figure 6, it can be seen that storage modulus of pure PMMA film is about 2000 MPa. Incorporation of a ZnS nanoparticle into PMMA film greatly increases its storage modulus—e.g., increasing to 3165 MPa as ZnS content increases to 70 wt %. However, pure PMMA film shows the highest amplitude of the damping peak ((tan δ)_max_) that decreases to 0.62 as the ZnS content increases to 70 wt %. Moreover, the glass transition temperature (T_g_), corresponding to the temperature of the maximum of tag δ, increases from 114 °C to 134 °C. These observations can be explained by improved cross-link density, indicating that more than one hydroxyl on the ZnS surface is grafted with polymerizable molecule, and polymerizable ZnS_0.15_ NPs act as a cross-linker in the nanocomposite films.

The loss spectra (tan δ versus temperature) of the ZnS/PMMA nanocomposite films decreases with improved ZnS content, and all the maximum of tan δ are less than 1, differing from pure PMMA film (the maximum of tag δ is about 1.48). This indicates that the distance between cross-link sites decreases [20], and that nanocomposite films have a higher storage modulus than loss modulus. The widths at the half-height on the relaxation peak are sensitive to the homogeneity of the networks. In Figure 6, it is clear that the nanocomposite films exhibited a single glass transition peak, indicating further uniform dispersion of ZnS in the nanocomposite films as expected.

**Figure 7:**
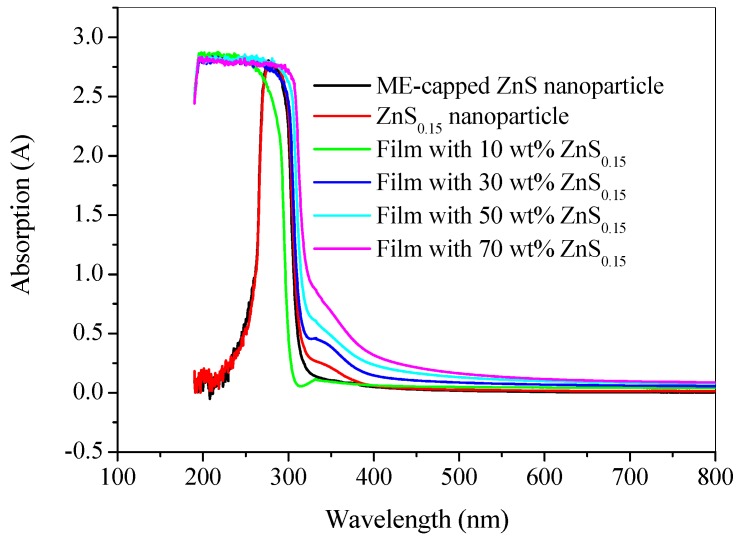
UV absorption spectra of ME-capped ZnS, polymerizable ZnS_0.15_ and ZnS/PMMA nanocomposite films with different ZnS_0.15_ content_._

The UV absorption of ME-capped ZnS, polymerizable ZnS_0.15_, and the nanocomposite films with different ZnS content is shown in Figure 7. It can be seen that ME-capped ZnS and polymerizable ZnS_0.15_ show a similar absorption spectrum, with a maximum absorption centre at 277 nm (4.47 eV), corresponding to a calculated ZnS nanoparticle diameter of about 3 nm, according to Brus’s model [14]. This value is similar to the particle size observed directly from TEM images shown in Figure 1. The excited state energy (4.47 eV) is larger than a band gap bulk value of 3.66 eV for ZnS. Such a strong and narrow absorption peak at 277 nm is known to arise from quantum confinement effect, which occurs when the particle size of nanocrystals is comparable with the Bohr radius. Polymerizable ZnS_0.15_ and ZnS/PMMA nanocomposite films show further absorption at 340 nm (3.66 eV), corresponding to the characteristic absorption peak of ZnS bulk material, and the intensity of the absorption increases as ZnS content in the nanocomposite film improves. This indicates that part of ZnS NPs may lose their quantum effect due to aggregation. The exited energy of the quantum dot is inversely related to its particle size [21]. The nanocomposite film with 10 wt % ZnS_0.15_ shows a slight blue shift compared to ME-capped ZnS and polymerizable ZnS_0.15_. This indicates that the incorporation of ZnS in the polymer by copolymerization stabilizes nanoparticle size by blocking the aggregation. The luminescent efficiency of the quantum dots increases with decreasing particle size, exhibiting better luminescent properties with smaller QD particle sizes [22]. The maximum absorption wavelength shows a slight redshift with ZnS_0.15_ increasing content in the nanocomposite films. This indicates that the particle size increases when high ZnS content is incorporated into the nanocomposite films.

**Figure 8:**
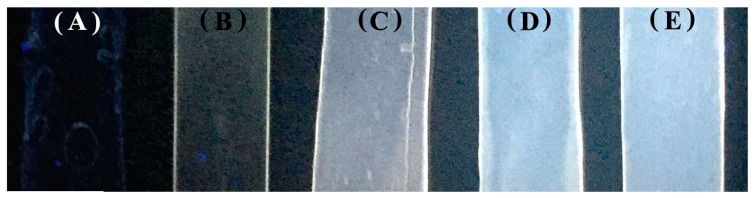
Fluorescence photographs of the ZnS/PMMA nanocomposite films with ZnS_0.15_ of (**A**) 0, (**B**) 10 wt %, (**C**) 30 wt %, (**D**) 50 wt %, and (**E**) 70 wt % under UV irradiation at a wavelength of 365 nm.

**Figure 9:**
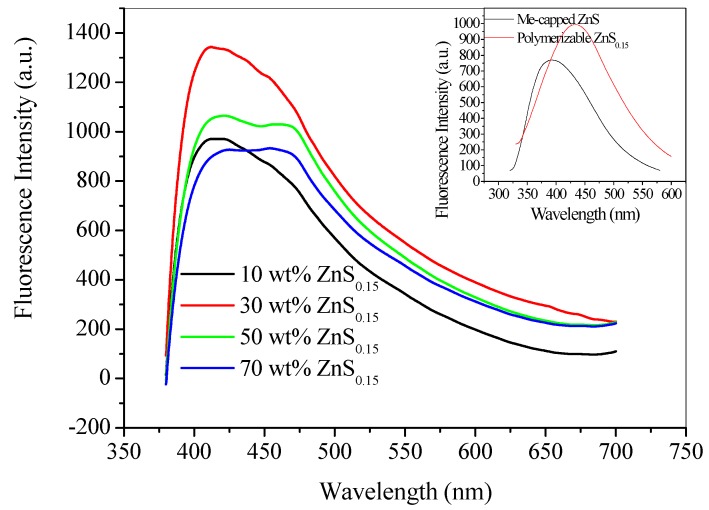
Photoluminescence emission spectra of ME-capped ZnS, polymerizable ZnS_0.15_, and nanocomposite films with different levels of ZnS content.

Figure 8 shows fluorescence photographs of the ZnS/PMMA nanocomposites with different ZnS_0.15_ content under UV irradiation at a wavelength of 365 nm. No obvious fluorescence was observed for pure PMMA film at an excitation wavelength of 365 nm. However, as a typical semiconductor, ZnS quantum dots possess interesting fluorescent characteristics that are preserved in the nanocomposite films as expected; the fluorescence intensity seemed to increase when the ZnS_0.15_ content increases from 0 to 50 wt %. In addition, we found that ZnS quantum dots are unstable, and their fluorescent intensity decreases greatly after one-day displacement away from light. However, no obvious decrease was observed for the fluorescent intensity of PMMA-based nanocomposite films. This indicates that ZnS is more stable in PMMA-based matrices than in DMF solution. The emission spectra of ME-capped ZnS and polymerizable ZnS_0.15_ dispersed in DMF with a concentration 10 mg/mL are shown as the insert in Figure 9. Usually, redshift in the peak emission wavelength occurs as the concentration of QDs increases due to self-reabsorption and Förster resonance energy transfer (FRET) among neighboring QDs, and becomes more significant as the interparticle distance decreases [23]. The peak emission wavelength (λ_m_ax) redshifts from 390 nm of ME-capped ZnS to 430 nm of polymerizable ZnS_0.15_, and polymerizable ZnS_0.15_ has higher fluorescence intensity than that of ME-capped ZnS. The mission behavior exhibits a well-defined excitonic emission feature, consistent with the results for highly luminescent ZnS NPs [24]. The redshift of the peak emission wavelength indicates that polymerizable ZnS_0.15_ is prone to self-reabsorption and FRET among the QDs, probably because of its different surface ligand. The greater fluorescent intensity of polymerizable ZnS_0.15_ may be from the annealing effect of the thermal grafting reaction of the polymerizable molecule on an ME-capped ZnS surface, as well as the coverage of surface defects with polymerizable molecule chains [25]. Figure 9 shows the fluorescence emission spectra of the ZnS/PMMA nanocomposite film with excitation at 365 nm. The peak wavelength of characteristic emissions at about 413 nm is observed for ZnS/PMMA nanocomposite films with 10 and 30 wt %, indicating a minor hypsochromic shift compared with polymerizable ZnS_0.15_ in DMF. This shift can be explained by an increase in the refractive index of the surrounding matrix [26]. ZnS/PMMA nanocomposite film with 30 wt % ZnS_0.15_ shows a higher emission intensity than that with 10 wt % ZnS_0.15_; the emission intensity then drops as the ZnS_0.15_ content increases—e.g., at 50 wt % and 70 wt %. Moreover, two emission peaks were observed upon the nanocomposite with 50 and 70 wt %, indicating that self-absorption happens at a high ZnS content.

## 4. Conclusions

ME-capped ZnS quantum dots were synthesized, after which a polymerizable molecule of 2-isocyanatoethyl methacrylate (IEM) was chemically grafted onto the ZnS surface, in order to synthesize polymerizable group-capped ZnS. Furthermore, transparent nanocomposite films with ultra-high-loading ZnS nanoparticles (up to 41 wt %, determined by TGA) were successfully fabricated by bulk thermo-curing copolymerization between the polymerizable group-capped ZnS and the monomer of MMA. TEM and XRD analysis indicate that ZnS NP dispersed uniformly in the nanocomposite films and remains in its original crystalline structure of ME and polymerizable group-capped ZnS. The TGA measurement displays that ZnS/PMMA nanocomposite films possess better thermal stability than a pure PMMA polymer. The UV absorption and transmittance measurement showed that the incorporation of ZnS in the nanocomposite films slightly decreased its transmittance. However, all nanocomposite films possess strong absorption when the wavelength is between 300 and 400 nm. Luminescent photographs show that ZnS/PMMA nanocomposite films exhibit good optical properties. The fluorescence intensity under UV irradiation increased as ZnS increased in increments to 30 wt %, and then decreased as the ZnC increased further to 70 wt % due to self-reabsorption.

## Figures and Tables

**Table 1:** Formulations of transparent poly(methyl methacrylate) (PMMA)-based nanocomposites.

Samples	The Free Content of ZnS_0.15_	ZnS_0.15_ (g)	Monomer of MMA (g)	Solvent of DMF(g)
1	10 wt %	0.05	0.45	0.3
2	30 wt %	0.15	0.35	0.3
3	50 wt %	0.25	0.25	0.3
4	70 wt %	0.35	0.15	0.3

**Table 2:** Weight loss at each decomposition step and the residue at 600 °C.

Samples	Temperature of the First Weight Loss (°C)	Weight Loss (wt%)	Temperature of the Second Weight Loss (°C)	Weight Loss (wt%)	The Weight of Residue at 600 °C
Pure PMMA	260–415	89.78	-	-	0
NC with 10 wt % ZnS	250–330	9.31	360–470	62.15	12
NC with 30 wt % ZnS	250–330	14.04	360–470	44.49	28.79
NC with 50 wt % ZnS	250–330	21.65	360–470	27.02	35.19
NC with 70 wt % ZnS	250–330	25.89	360–470	20.74	40.96
Polymerizable ZnS_0.15_	250–330	24.28	-	-	62.95
ME-capped ZnS	250–330	24.64	-	-	63.47
